# Characteristics and pathogenicity of *Vibrio alginolyticus* SWS causing high mortality in mud crab (*Scylla serrata*) aquaculture in Hong Kong

**DOI:** 10.3389/fcimb.2024.1425104

**Published:** 2024-07-23

**Authors:** Carsten Tsun-Ka Kwok, Ray Chun-Wai Yu, Pak-Ting Hau, Karry Yuen-Ching Cheung, Iain Chi-Fung Ng, Joshua Fung, Ivan Tak-Fai Wong, Miranda Chong-Yee Yau, Wai-Man Liu, Hang-Kin Kong, Gilman Kit-Hang Siu, Franklin Wang-Ngai Chow, Sai-Wang Seto

**Affiliations:** ^1^ Department of Food Science and Nutrition, The Hong Kong Polytechnic University, Hong Kong, Hong Kong SAR, China; ^2^ Department of Health Technology and Informatics, The Hong Kong Polytechnic University, Hong Kong, Hong Kong SAR, China; ^3^ Research Centre for Chinese Medicine Innovation, The Hong Kong Polytechnic University, Hong Kong, Hong Kong SAR, China; ^4^ Department of Clinical Pathology, Pamela Youde Nethersole Eastern Hospital, Hong Kong, Hong Kong SAR, China; ^5^ AI Farming Company (Hong Kong) Limited, Hong Kong, Hong Kong SAR, China; ^6^ Research Institute for Future Food, The Hong Kong Polytechnic University, Hong Kong, Hong Kong SAR, China; ^7^ NICM Health Research Institute, Western Sydney University, Penrith, NSW, Australia

**Keywords:** pathogen, *Vibrio alginolyticus*, mud crab, *Scylla serrata*, disease, histopathology, infection, aquaculture

## Abstract

**Introduction:**

*Vibrio alginolyticus* is a Gram-negative, rod-shaped bacterium belonging to the family of *Vibrionaceae*, a common pathogen in aquaculture animals, However, studies on its impact on *Scylla serrata* (mud crabs) are limited. In this study, we isolated *V. alginolyticus* SWS from dead mud crab during a disease outbreak in a Hong Kong aquaculture farm, which caused up to 70% mortality during summer.

**Methods:**

Experimental infection and histopathology were used to investigate the pathogenicity of *V. alginolyticus* SWS in *S. serrata* and validate Koch’s postulates. Comprehensive whole-genome analysis and phylogenetic analysis antimicrobial susceptibility testing, and biochemical characterization were also performed.

**Results:**

Our findings showed that *V. alginolyticus* SWS caused high mortality (75%) in *S. serrata* with infected individuals exhibiting inactivity, loss of appetite, decolored and darkened hepatopancreas, gills, and opaque muscle in the claw. Histopathological analysis revealed tissue damage and degeneration in the hepatopancreas, gills, and claw muscle suggesting direct and indirect impacts of *V. alginolyticus* SWS infection.

**Conclusions:**

This study provides a comprehensive characterization of *V. alginolyticus* SWS as an emerging pathogen in *S. serrata* aquaculture. Our findings underscore the importance of ongoing surveillance, early detection, and the development of targeted disease management strategies to mitigate the economic impact of vibriosis outbreaks in mud crab aquaculture.

## 1 Introduction

Mud crabs is a collective term referring to four different crabs’ species, namely, *Scylla serrata*, *Scylla olivacea*, *Scylla paramamosain*, and *Scylla tranquebarica*, which belong to the family Portunidae (Fazhan et al., 2017). Among these species, *S. serrata* is the most widely distributed species of mud crabs and is commonly cultivated (Paran et al., 2022). They inhabit estuaries and mangrove areas and are regarded as one of the most popular and valuable seafood globally due to their nutritional value, palatability, and high market demand (Bhuiyan et al., 2021). In recent decades, the demand for mud crabs has continuously increased in the world, coinciding with a decline in natural population due to overfishing. Hence, aquaculture has become an increasingly prevalent means of meeting the global demand, with worldwide production of mud crabs rising from 10.7 kt in 2000 to 248.8 kt in 2020 (FAO, 2022). While the aquaculture industry for mud crabs is expected to continue growing in response to the market demand, sustainability and profitability are hindered by high mortality rate due to cannibalism and diseases (Alberts-Hubatsch et al., 2016; Ly Van et al., 2022). The management of disease outbreak in *Scylla* spp. aquaculture remains a challenging task for the industry; the emergence of new diseases, highly contagious characteristics, and pathogen resistance to drugs can lead to substantial economic loss (Hu et al., 2021; Apine et al., 2023). Therefore, it is crucial to study the pathogenicity and profile of pathogens in *Scylla* spp. especially in aquaculture.

Currently, there are approximately 30 documented infectious diseases found to afflict mud crab caused by bacteria, viruses, parasites, and fungi (Jithendran et al., 2011). Among them, viral and bacterial infections are the most common with typically pathogens, such as the white spot syndrome virus and chitin-digesting bacteria, including *Vibrio* spp., *Aeromonas* spp., *Pseudomonas* spp., and *Spirillium* spp (Jithendran et al., 2011). Notably, several *Vibrio* species, including *V. vulnificus*, *V. splendidus*, *V. orientalis*, *V. alginolyticus*, *V. harveyi*, and *V. parahemolyticus*, have been reported to cause high mortality in *Scylla* spp. due to shell disease and milky disease (Lavilla-Pitogo et al., 2001; Sarjito et al., 2018; Gunasekaran et al., 2019; Cheng et al., 2020). However, while most studies investigating diseases of mud crab focus on *V. parahemolyticus* and white spot syndrome virus, the pathogenicity of *V. alginolyticus* in mud crab, particularly *S. serrata*, remains underexplored.

*V. alginolyticus* is a species of Gram-negative, rod-shaped bacteria belonging to the family *Vibrionaceae*. It is the second most common *Vibrio* species found in coastal, marine, and aquatic environments (Yu et al., 2022). Increasing number of studies have reported that *V. alginolyticus* is a pathogen affecting a variety of aquatic animals, including fish, shrimp, and oyster, resulting in high mortality and substantial economic loss in aquaculture (George et al., 2005; Cao et al., 2018; Yang et al., 2021). *V. alginolyticus* is also well known to cause seafood poisoning and systemic infection in humans (Li et al., 2009; Hong et al., 2014). However, in the context of mud crab, *V. alginolyticus* has only been recognized for causing shell disease, and the pathogenicity and characteristics of *V. alginolyticus* isolated from *S. serrata* (mud crab) have not been well studied (Gunasekaran et al., 2019).

Recently, *S. serrata* aquaculture in Hong Kong experienced a significant mortality rate, with up to 70% of crabs dying during the summer period due to unknown causes. Site investigations found that all of the water parameters on the farm were normal, and the use of antibiotics was strictly prohibited according to farm practices. Based on these observations, we hypothesized that the outbreak might be caused by a bacterial agent. In this study, pure *V. alginolyticus* SWS was isolated from a crab that died on the aquaculture. Experimental infection was then conducted on *S. serrata* to assess its impact on mortality, pathogenicity, and disease manifestation. In addition, to characterize this strain of bacterium, we performed whole-genome sequencing (WGS), biochemical tests, and antibiotic susceptibility test (AST). Results from our crab infection model indicate that *V. alginolyticus* SWS is a strain of deadly pathogen to *S. serrata* causing infected crabs to become inactive, lose appetite, and lose normal appearance of the muscle, gills, and hepatopancreas, and induce tissue damage and degeneration in the hepatopancreas, gills, and claw muscle in the infected crab. Our current study provides useful information for disease surveillance and formulation of effective disease prevention measurements in mud crab aquaculture.

## 2 Materials and methods

### 2.1 Bacterial isolation

*Vibrio* species were isolated from two diseased *S. serrata* supplied by AI Farming Company (Hong Kong) Limited. Samples were swabbed from the inner shell of the diseased mud crab into sterilized phosphate-buffered saline (PBS) followed by streaking on LB agar with 3% NaCl and incubated at 37°C for 24 h. After 24h, a single colony was picked and further streaked on trypticase soy agar (TSA) with 8% NaCl at 37°C for 24 h. Eventually, a single colony was picked and further streaked on thiosulfate–citrate–bile salt–sucrose (TCBS) agar. The pure isolates were either routinely sub-cultured for sequent analysis or stored at −80°C in tryptic soy broth (TSB) with an 8% salt supplement containing 15% glycerol. The isolated colonies from two disease crabs were tentatively identified by mass spectrometry matrix-assisted laser desorption ionization-time of flight (MALDI-TOF) mass spectrometry system (Microflex LT with MALDI Biotyper Sirius one RUO system, Bruker Daltonics, Bremen, Germany) equipped with FlexControl software, version 3.3 (Bruker Daltonics) MALDI‐TOF (2.4.2), and their morphology was also observed.

### 2.2 Experimental infection

Eighteen healthy mud crab *S. serrata* (112.5 ± 17.7 g) were kindly provided by AI Farming Company (Hong Kong) Limited. Crabs were maintained in 22–23 ppt (part per thousand) brackish water with 6.8–7.2 pH at 26 ± 2°C in AI farming. Before challenging with bacterial isolate, crabs were acclimatized in 23 ppt brackish water for 30 min. The crabs were divided into two groups, the infection group, and the control group, consisting of 8 and 10 mud crabs, respectively. They were maintained in two plastic tanks (100 L, 117 × 43 × 20 cm^3^) with 70 L of filtered artificial seawater at 21.2–23 ppt salinity at 23°C, proper aeration, and 12h light/dark cycle. Crabs were fed with commercial pellet feed and fish meat, approximately 10% of their weight. The infection group was injected with 500 μl of bacterial suspension in sterile 0.85% saline with a final concentration of 2.5 × 10^5^ CFU/ml at the base of their right fourth walking leg using a 25G needle. Similarly, the control group was injected with 500 μl of 0.85% sterile saline. The mortalities and symptoms were monitored daily for 7 days. *V. alginolyticus* SWS was re-isolated from the inner crab shell and hemolymph in infected crab, but not found in healthy control crab, which meets the criteria of Koch’s postulates. Hepatopancreases, gills, and muscles from claws were collected for histology.

### 2.3 Histopathological examination

The tissues (hepatopancreas, gill, and claw muscle) of infected crabs and a control crab were subjected to routine crustacean tissue-processing procedures adapted from “Histological techniques for marine bivalve mollusks and crustaceans, second edition” (Howard, 2004). In short, the tissues collected were first fixed in 40% formalin followed by storage in 75% ethanol. Then, the tissues were dissected to an appropriate thickness of approximately 0.5 cm and subjected to automated tissue processing under vacuum using Excelsior AS Tissue Processor (Epredia). During the tissue processing, the tissues were dehydrated by a series of graded ethanol, cleared by three changes of xylene, and infiltrated with three changes of paraffin wax, followed by manual embedding. The blocks were sectioned at 6 µm, and then hematoxylin and eosin (H&E) staining was performed on the sections to visualize histological details under light microscopy using Eclipse Ci Microscope (Nikon) with different magnifications (×100, ×200, and ×400).

### 2.4 Molecular biological species identification

#### 2.4.1 Bacterial genomic DNA extraction and purification

Prior to genomic DNA (gDNA) extraction for WGS, four single colonies from each TCBS agars were picked to have tentative identification using MALDI Biotyper (RUO) MALDI-TOF Mass Spectrometry Based Microorganism Identification System (Bruker Daltonics, USA). After that, tentative identified single colonies were picked, and Genomic DNA was extracted and purified from it using a QIAamp^®^ BiOstic^®^ Bacteremia DNA kit (Qiagen, Valencia, CA) with reference to the manufacturer’s protocol. The concentration of the purified genomic DNA was determined using a NanoDrop One Microvolume UV-Vis Spectrophotometer (Thermo Scientific, USA). All DNA was stored at −80°C prior to further analysis.

#### 2.4.2 Tentative identification using mass spectrometry MALDI‐TOF

For bacterial identification, colonies freshly picked from TCBS agar, isolated from deceased mud crabs, were utilized. Each colony was mixed with 1 µl of matrix solution and subsequently dried on a stainless-steel target plate. Identification was performed using MALDI-TOF. The acquired spectra were analyzed and compared against the Bruker Biotyper database using the MBT Compass Library (version 12.0.0.0).

#### 2.4.3 Genome sequencing and bioinformatics analysis (annotation of genome assembly)

Bacterial whole-genome sequencing of *V. alginolyticus* SWS was sent to Azenta Life Sciences to perform sequencing by Illumina NovaSeq 6000 in PE150 mode. The Raw DNA-Seq data were deposited to the US National Center for Biotechnology Information (NCBI) database under accession number PRJNA1101690. For construction of complete-genome sequence, the qualified DNA reads were assembled using the Assembly RAST (ARAST) service with auto mode in Pathosystems Resource Integration Center (PATRIC) server comprehensive genome analysis function (Wattam et al., 2016). The genome Annotation was performed by Rapid Annotation using Subsystem Technology (RAST), which is annotated using genetic code 11. Subsystem Analysis of the genomic was also performed using PATRIC. Specialty genes, including transporter virulence factors, drug targets, and antibiotic resistance genes were also annotated with reference to specific source databases using PATRIC. Antimicrobial resistance (AMR) genes was also predicted by utilizing k-mer-based AMR gene detection method in PATRIC, while a virulence factors database (http://www.mgc.ac.cn/VFs/main.htm) was used to predict the virulence factors in *V. alginolyticus* SWS (Liu et al., 2019, 2022). Phylogenetic analysis was also performed with the aid of PATRIC that selects the closest reference and representative genomes by Mash/MinHash algorithm; PATRIC global protein families were selected to determine the phylogenetic placement of the isolated genome. The protein sequences were aligned with MUSCLE algorithm (multiple sequence alignment with high accuracy and high throughput), and the nucleotides for each of those sequences were mapped to the protein alignment. The joint set of amino acid and nucleotide alignments were concatenated into a data matrix, and Randomized Axelerated Maximum Likelihood (RaxML) was used to analyze the matrix with fast bootstrapping method. Genome similarity was studied by calculating the pairwise average nucleotide identity (ANI) based on BLAST+ (ANIb) and MUMmer (ANIm) using JSpeciesWS (version 4.1.1, https://jspecies.ribohost.com/jspeciesws/#home) (Richter et al., 2015).

### 2.5 Characterization of isolated bacteria

#### 2.5.1 Salt tolerance test

A single colony of *V. alginolyticus* SWS was picked from freshly streaked TCBS and inoculated into nutrient broth (NB) supplementary with different percentages of salt, 0%, 1%, 6%, and 8% at 37°C, for 24 h for evaluating the salt requirements of crab isolate.

#### 2.5.2 Antimicrobial susceptibilities

Antibiotic susceptibility testing was conducted on *V. alginolyticus* SWS using the disk diffusion method, as per the guidelines established by the Clinical and Laboratory Standards Institute (CLSI). The test employed commercially available antibiotic disks from Oxoid (UK) and Liofilchem (Italy). A fresh isolate from crab was prepared by inoculation into 0.85% saline solution to match a 0.5-McFarland standard turbidity. This suspension was uniformly streaked onto Mueller–Hinton agar plates. Subsequently, antibiotic discs were applied to the inoculated plates. The antibiotics tested included cefoxitin (FOX, 30 µg), ceftriaxone (CRO, 30 µg), cefepime (FEP, 30 µg), ampicillin (AMP,10 µg), chloramphenicol (C, 30 µg), ciprofloxacin (CIP, 5 µg), colistin (CS, 10 µg), gentamycin (CN, 10 µg), nalidixic acid (NA, 30 µg), meropenem (MEM, 10 µg), imipenem (IMP, 10 µg), ertapenem (EPT, 10 µg), doripenem (DOR, 10 µg), tetracyclin (TE, 30 µg), trimethoprim/sulfamethoxazole (SXT, 25 µg), cefotaxime (CTX, 30µg), cefotaxime + clavulanic acid (CTL, 30 + 10 = 40 µg), ceftazidime (CAZ) (30 µg), ceftazidime + clavulanic acid (CAL, 30 + 10 = 40 µg), and amoxicillin–clavulanic acid (AUG/AMC, 30 µg). The inoculated plates were incubated at 35 ± 2°C for 16–18 h. Post-incubation, zones of inhibition were measured and interpreted using the BIOMIC V3 Microbiology System (Giles Scientific, USA), with reference values from both CLSI and the European Committee on Antimicrobial Susceptibility Testing (EUCAST).

#### 2.5.3 Metabolic/biochemical characterization

Vitek 2 Compact (bioMérieux. Marcy l’Etoile, France) Gram-Negative Identification (GNI) cards were used to determine the metabolic/biochemical characterization of crab isolate with reference to the manufacturer’s guideline. In brief, three to four fresh colonies were suspended in sterilized 0.85% saline and thoroughly mixed to obtain 0.5 McFarland standard. A 5-μl suspension of crab isolate was loaded into Vitek 2 GNI cards to perform 48 biochemical tests. The VITEK-2 system reported the results automatically with installed version 9.02.3.

#### 2.5.4 Optimal temperature and pH

The optimal growth condition of *V. alginolyticus* SWS, in terms of temperature and pH, was determined using the method of Norfolks et al. with slight adjustment (Norfolk et al., 2023). The isolate was subcultured from the TSB stored at –80°C (Section 2.1) at least twice. Then, a single colony of the isolate was inoculated into approximately 5 ml of LB broth with 3% NaCl (w/v) (LBS), followed by a 4h shaking incubation at 230 rpm and 37°C to reach stationary phases. After that, the culture was diluted by 10-fold to OD600 of approximately 0.1. Then, 2 µl of the culture was added to 148 µl of LBS with pH adjusted to 4, 5, 6, 7, 8, 9, and 10 ( ± 0.1) in a 96-well plate. The bacterial growth at different pH values under 26°C, 30°C, and 37°C was measured in terms of OD600 at regular intervals of 15 min for 14 h. The readings of OD600 were subjected to analysis using Dashing Growth Curves (Reiter and Vorholt, 2024). The readings were blanked using the average OD600 values of three wells with 150 µl of LBS at each temperature. The tight logistic model was used for growth curve fitting and computational determination of the exponential phase, while the growth curve was smoothed using rolling windows of 10. The application returned the doubling time for each well, which was then used to determine the optimal growth condition.

## 3 Results

### 3.1 Clinical signs of the naturally infected mud crab from a local crab farm

**Figure 1:**
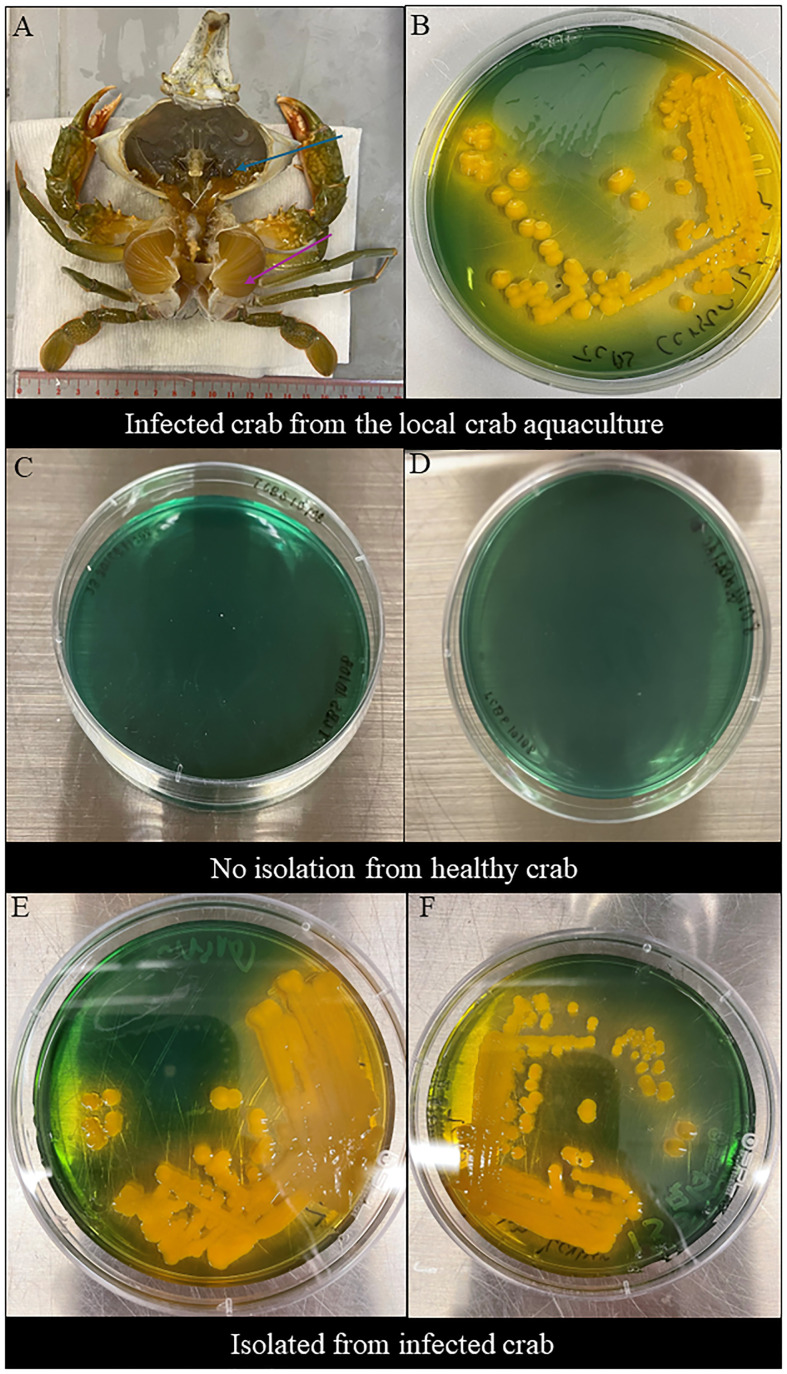
Clinical sign of infected mud crab and morphological observations in TCBS agar. **(A)** Clinical sign of naturally infected *S. serrata* from the mud crab farm with decolorized darkened hepatopancreas with flaccid and atrophied appearance (blue arrow) and decolorized gills (purple arrow). **(B)** Morphological observations of *V. alginolyticus* SWS isolated from infected *Scylla serrata* from the local crab aquaculture formed large, round, mucoid, convex, yellow colonies in TCBS. **(C, D)** No *Vibrio* isolated from the inner crab shell and hemolymph of healthy crabs. **(E, F)** Morphological observations of *V. alginolyticus* SWS reisolated from the inner crab shell in TCBS agar showed the formation of large, round, mucoid, convex, yellow colonies **(E)** and hemolymph **(F)** of *V. alginolyticus* SWS-infected crabs.

The clinical signs of infected *S. serrata* from aquaculture were observed after dissection. Infected *S. serrata* had a darkened hepatopancreas with flaccid and atrophied appearance and decolorized gills (**Figure 1A**).

### 3.2 Morphological observation of isolated bacteria and tentative identification

*V. alginolyticus* SWS formed large, round, mucoid, convex, yellow colonies on TCBS agar (**Figure 1B**), which indicated that they could ferment sucrose.

The tentative identification of this *V. alginolyticus* SWS was performed by MALDI-TOF. Results revealed that the bacteria isolated from two dead crabs probably were *Vibrio* genus, although the species identification was identified as *V. alginolyticus*. The score value was arranged from 1.82 to 1.99 implying the species identification is in low confidence, which required a method with higher differentiating power (Mougin et al., 2020).

Swabs were retrieved from the inner shell of infected and healthy mud crab and their hemolymph to isolate bacteria. No bacteria were isolated from healthy crabs (**Figures 1C, D**), while *V. alginolyticus* SWS were again isolated from experimentally infected crabs (**Figures 1E, F**). These imply that the bacteria used in the infection experiment was the causative infectious agents and met the criteria of Koch’s postulates suggesting that *V. alginolyticus* SWS is a pathogen to *S. serrata*.

### 3.3 *V. alginolyticus* SWS in *S. serrata* infection model

#### 3.3.1 Mud crab survival

**Figure 2:**
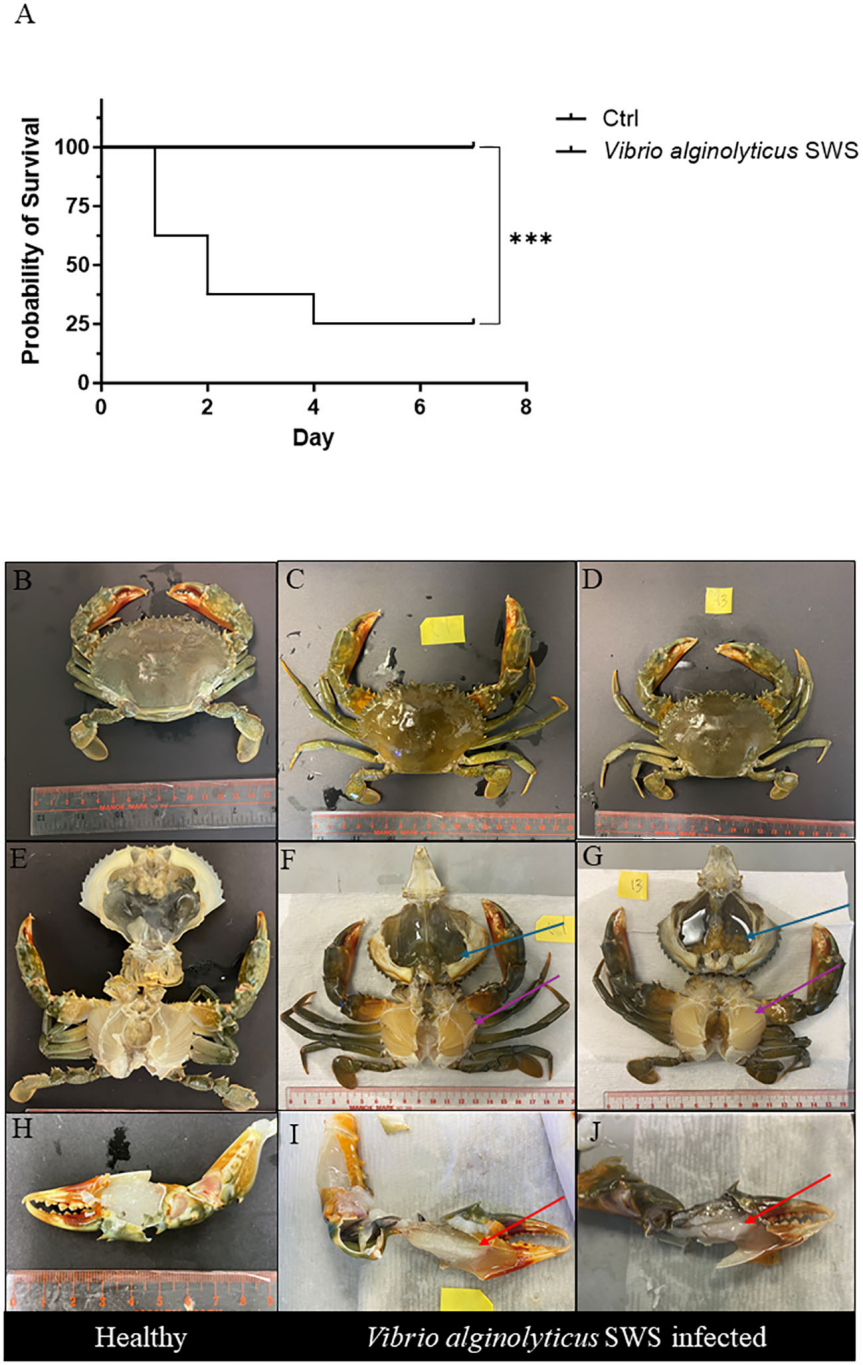
Effect of *Vibrio alginolyticus SWS* on survival and induced symptoms in *S. serrata*. **(A)** Survival curve of mud crab in the experimental infection of *V. alginolyticus* SWS (infected group = 8, control = 10); log-rank (Mantel–Cox) test was used to compared the difference between two groups, ***p < 0.001 versus control. **(B–D)** Comparison between the exoskeleton of healthy mud crab **(B)** and *V. alginolyticus* SWS-infected mud crab **(C, D)**. **(E)** Healthy crab without clinical signs. **(F, G)** *V. alginolyticus* SWS-infected *S. serrata* with decolorized darkened hepatopancreas with flaccid and atrophied appearance (blue arrow) and decolorized gills (purple arrow). **(H)** Healthy *S. serrata* with a transparent white muscle in the claw. **(I, J)** *V. alginolyticus* SWS-infected *S. serrata* with opaque white muscle in the claw (red arrow).

The 7 days survival curve of mud crabs challenged with or without *V. alginolyticus* SWS is illustrated in **Figure 2A**. Throughout the experimental infections, the control/mock crab (PBS group) remained healthy, while the infected crab began to die after 1 day of *V. alginolyticus* SWS injection and reached a 75% cumulative mortality rate on day 4 (**Figure 2A**). This suggested that *V. alginolyticus* SWS was highly pathogenic to *S. serrata*.

#### 3.3.2 Clinical signs of diseased mud crab

Infected *S. serrata* was monitored daily; visual inspection on exoskeleton and necropsy was carried on infected mud crab and healthy mud crab to observe lesions. Infected crabs had low appetite and were inactive before their death. There was no obvious lesion on the external shell of infected mud crabs (**Figures 2C–F**) and control crabs (**Figure 2B**). In contrast, morphological observations from necropsy of both healthy crabs (**Figure 2E**) and infected crabs (**Figures 2F, G**) showed that infected crabs had decolorized and darkened hepatopancreas with flaccid and atrophied appearance (dark green, **Figure 2F**; pale brown, **Figure 2G**) and decolorized gills (**Figures 2F, G**). When comparing the muscle in the claw of healthy crabs (**Figure 2H**), infected crabs had an opaque muscle with milky appearance (**Figures 2I, J**).

#### 3.3.3 Histopathology of infected *S. serrata*

**Figure 3:**
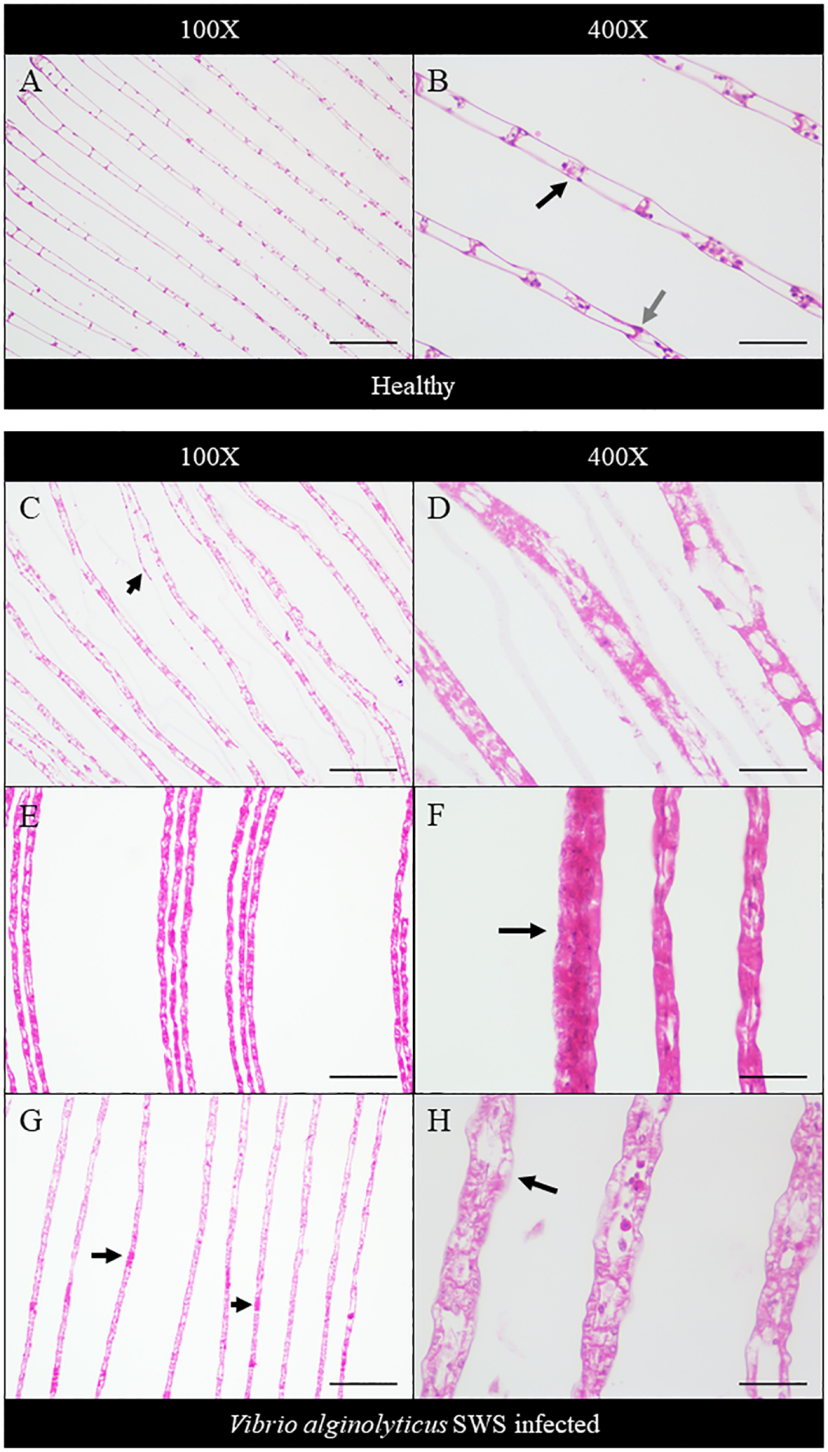
Histopathological analysis of the gills under ×100 **(A, C, E, G)** and ×400 magnification **(B, D, F, H)**. In the control crabs, basic lamellae structures were seen **(A)**, with the presence of hemocyte nodules (black arrow) in the cavity of the gills and spindle-shaped nuclei of epithelial cells (gray arrow) **(B)**. In heavily infected crabs, the lamellae structure of the gills was destroyed with exfoliated epithelial cells (black arrow) **(C)**, and eosinophilia was seen with much cell debris and rounded nuclei of epithelial cells **(D)**. In moderately infected crabs, the lamellae start to degenerate with gross eosinophilia **(E)**, while heavy infiltration of hemocytes was also seen (black arrow) **(F)**. In less-infected crabs, the lamellae remained rather intact with the presence of eosinophilic masses within the cavity (black arrows) **(G)**, and vacuolation occurred in the epithelial cells (black arrow), with leakage of cell debris into the cavities, which were still observable **(H)**. Scale bars represented 200 µm in ×100 magnification and 50 µm in ×400 magnification, respectively.

Histopathology was performed to study the impact of *V. alginolyticus* SWS on *S. serrata*. The impact of *V. alginolyticus* SWS infection on the gills of crabs was obvious. In healthy crabs, the lamellae structures of the gills are well organized (**Figure 3A**). The lamellae consist of nodules of hemocytes in the gill cavities and thin epithelial cells enclosing the cavities (**Figure 3B**). In the crabs with most severe tissue damage, the basic lamellae structures of the gills were destroyed (**Figure 3C**). On a higher magnification (×400), necrotic features were found with eosinophilia and plenty of cell debris. The spindle-shaped nuclei of epithelial cells are rounded (**Figure 3D**). Hemocytes, which present as a group of few inside the gill cavity normally, may heavily infiltrate into the gill cavities of infected crabs (**Figure 3F**). The histological damage was weaker in some other crabs. The lamellae may still degenerate, but to a smaller extent (**Figures 3E, G**). Vacuolation may occur in the epithelial cells. The cavities, though observable, started to fill with cell debris (**Figure 3H**).

**Figure 4:**
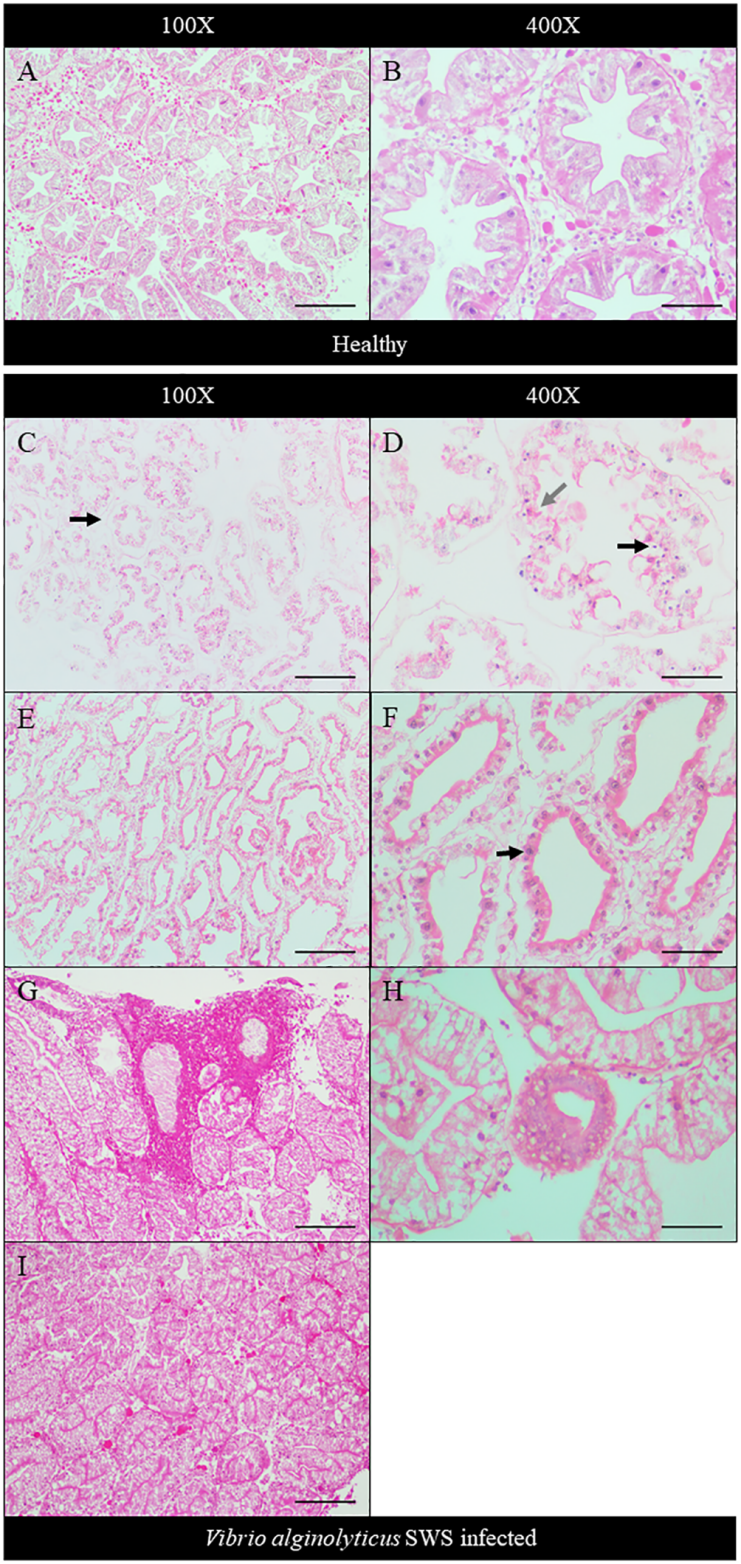
Histopathological analysis of hepatopancreas under ×100 **(A, C, E, G, I)** and ×400 magnification **(B, D, F, H)**. In control crabs, tubular structure and star-shaped lumen were present with interstitial tissue filling between tubules **(A, B)**. In heavily infected crabs, the tubular degenerated with the detachment of cells from the basement membrane (black arrow) and no observable interstitial tissues **(C)**, while severe cell necrosis was seen with pyknotic nuclei (black arrow), eosinophilic and fragmented cytoplasm (gray arrow), and indistinguishable cell boundaries **(D)**. In moderately infected crabs, the tubule degeneration was milder with widened lumen and shrinkage of tubular cells **(E)**. Strong eosinophilia was found in tubular cells, with chromatin margination and prominent nucleoli seen (black arrow) **(F)**. In less-infected crab, heavy infiltration of hemocytes was seen **(G)**. Granuloma-like structure was observed **(H)**, and the tubular lumen shrank or even fused **(I)**. Scale bars represented 200 µm in ×100 magnification and 50 µm in ×400 magnification, respectively.

The extent of hepatopancreatic degeneration also varied among crabs. The hepatopancreas of healthy crabs demonstrated round tubular structures with star-shaped lumens (**Figure 4A**). The spaces between the tubules are filled with interstitial tissues (**Figure 4B**). Severe necrosis of hepatopancreas was observed with the loss of its characteristic tubular structure. Tubular cells detached, and the lumen widened (**Figure 4C**). On the cellular level, nuclear pyknosis occurred with eosinophilic and fragmented cytoplasm. Together with the indistinguishable cell boundaries, all of these showed typical necrotic features (**Figure 4D**). In some other crabs, the tubular degeneration became milder, with cells presenting inflammatory response (**Figures 4E, F**). Also, heavy infiltration of hemocytes was found in the hepatopancreas of another crab (**Figure 4G**). The tubular lumen was fused (**Figure 4H**), and a granuloma-like structure was found (**Figure 4I**).

**Figure 5:**
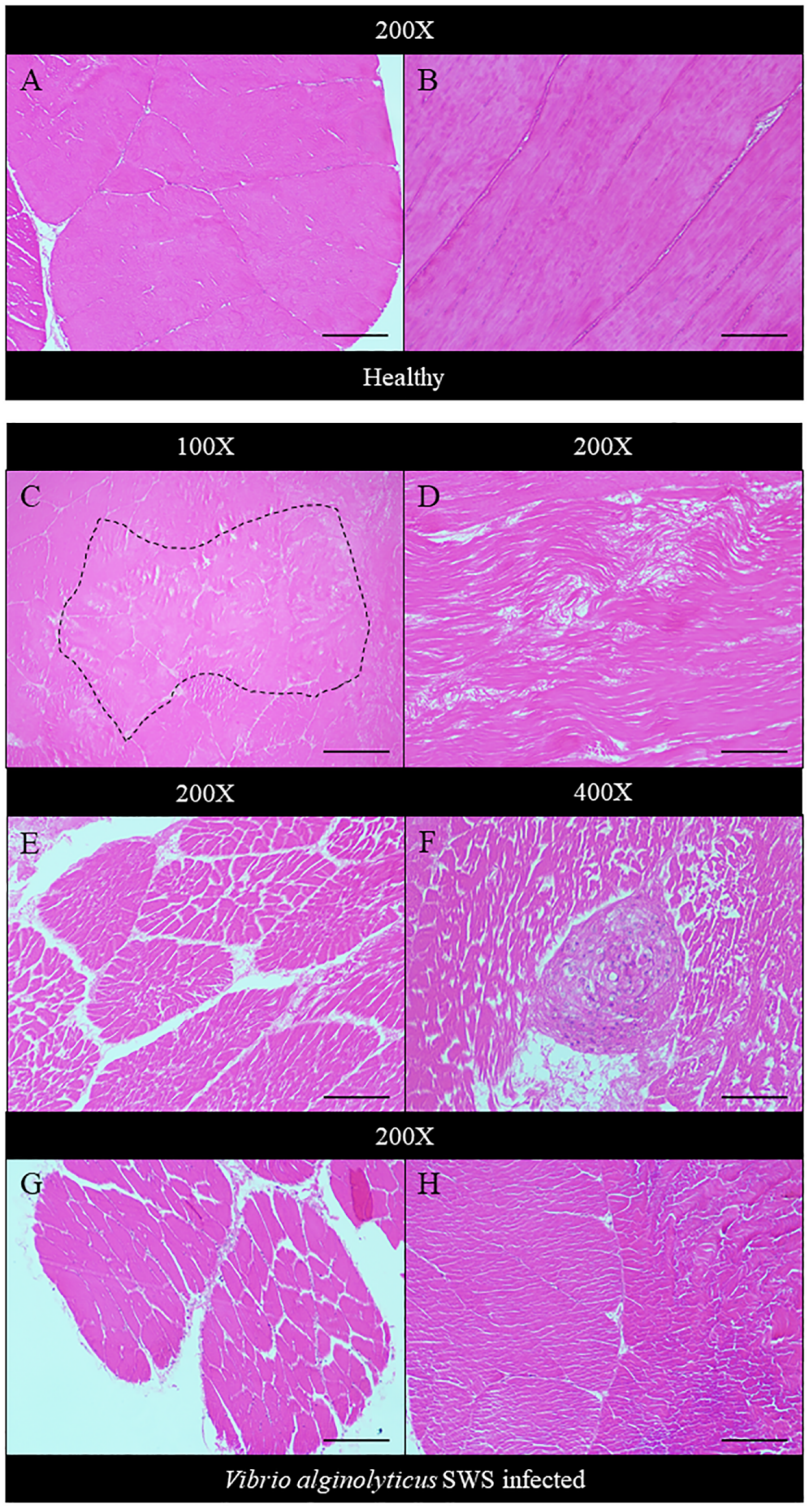
Histopathological analysis of muscle under ×100 **(C)**, ×200 **(A, B, D, E, G, H)**, and ×400 magnification **(F)**. In control crabs, muscle fibers were tightly packed and well organized in transverse **(A)** and longitudinal sections **(B)**. In heavily infected crabs, an amorphous mass of muscle tissue was seen (dotted box) **(C)**, and muscle fiber derangement occurs with disrupted organization **(D)**. Muscular atrophy started to occur with gaps present between fibers of a single muscle bundle **(E)**. A granuloma-like structure was also seen **(F)**. The extent of muscular atrophy decreases in moderately infected **(G)** and least infected crabs **(H)**. Scale bars represented 200 µm in ×100 magnification, 100 µm in ×200 magnification, and 50 µm in ×400 magnification.

In the infected crabs, degeneration of muscles also occurred. Compared to the healthy muscle of crabs which showed tightly packed muscle fibers (**Figures 5A, B**), both the transverse and longitudinal organizations of muscle fibers were affected. Muscular atrophy was found in the tissues of infected crabs. The normal structure of muscles was disrupted with amorphous masses and derangement of muscle fibers seen (**Figures 5C–H**). A granuloma-like tissue was also found (**Figure 5F**).

### 3.4 Molecular identification and annotation of genome

#### 3.4.1 Comprehensive genome analysis and annotation of genome assembly

Comprehensive genome analysis service at PATRIC was used to assemble the genome of *V. alginolyticus* SWS. The assembled genome features of *V. alginolyticus* SWS are summarized in **Supplementary Table 1**. In brief, the assembled genome of *V. alginolyticus* SWS had 87 Contigs and 44.61% average G + C content with an estimated genome length of 5,215,511 bp.

Annotation of the genome of *V. alginolyticus* SWS was done by performing RAST tool kit in PATRIC. The results showed that the genome of *V. alginolyticus* SWS had 4,876 protein coding sequences (CDS), 78 transfer RNA (tRNA) genes, and 3 ribosomal RNA (rRNA) genes (**Supplementary Table 2**). The annotation further revealed that the protein features of *V. alginolyticus* SWS include 1,128 hypothetical proteins and 3,748 proteins with functional assignments (**Supplementary Table 3**). Among proteins that had functional assignments, 1,087 had Enzyme Commission (EC) number assignments, 910 with gene ontology (GO) assignments, and 802 with gene and genome pathway mapping. In addition, the antimicrobial resistance (AMR) genes annotated and their corresponding mechanism are summarized in **Supplementary Table 3**.The annotated genome of *V. alginolyticus* SWS is plotted as a circular graph (**Supplementary Figure 1A**), and the subsystem analysis of the genomes showed that majority of genes were linked to subsystem (a group of proteins that create structural complex or work together to carry out a specific biological activity) related to metabolism, stress response, defense, virulence, and protein processing (**Supplementary Figure 1B**). Virulence factor (VF) database revealed that *V. alginolyticus* SWS might possess 151 VF-related genes from different classes of VF including adherence, anti-phagocytosis, chemotaxis and motility, iron uptake and quorum sensing, secretion system, toxin, and biofilm formation (**Supplementary Table 4**).

#### 3.4.2 Phylogenetic analysis and genome similarity

**Figure 6:**
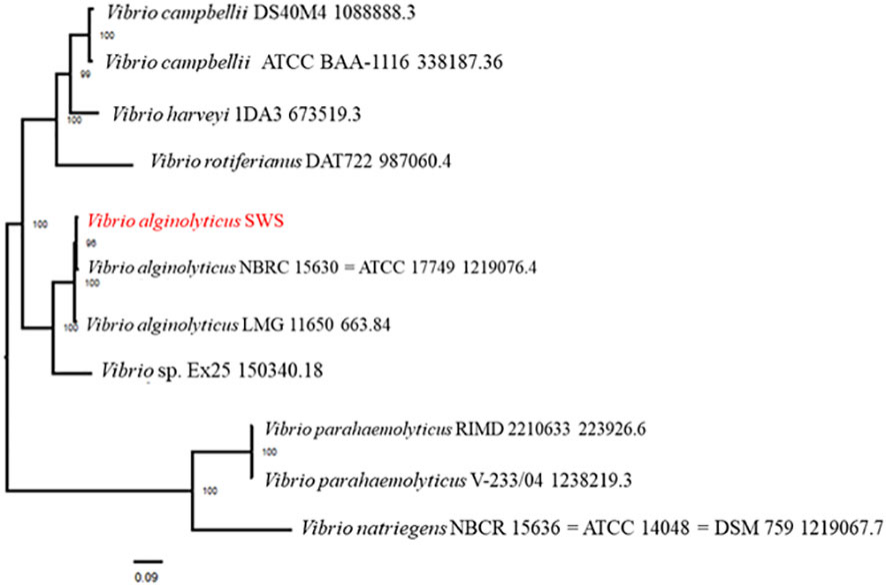
Phylogenetic analysis of *Vibrio alginolyticus* SWS. *V. alginolyticus* SWS was clustered to *V. alginolyticus* ATCC 17749.

Phylogenetic analysis of *V. alginolyticus* SWS was performed in PATRIC service by comparing PATRIC global protein families from genomes of closest reference and representative genomes identified by Mash/MinHash. The result of phylogenetic analysis showed that *V. alginolyticus* SWS clustered with *V. alginolyticus* NBRC 15630 (ATCC 17749) (**Figure 6**), thus confirming the species identification of the isolate as *V. alginolyticus*. Genome similarity of *V. alginolyticus* SWS among 15 V*. alginolyticus* species was calculated by ANIb and ANIm utilizing JSpeciesWS. **Supplementary Table 5** shows that *V. alginolyticus* SWS and the other 15 V*. alginolyticus* had ANI values higher than 98% suggesting they were highly similar.

### 3.5 Characterization of the *V. alginolyticus* SWS

#### 3.5.1 Salt-tolerant *V. alginolyticus* SWS

*V. alginolyticus* SWS was inoculated into nutrient broth (NB) with different NaCl % to investigate its salt tolerance characteristics. It was unable to grow in NB with no salt; in contrast, *V. alginolyticus* SWS grew in NB ranging from 1% to 8% NaCl (**Table 1**), where *V. alginolyticus* SWS grow much better in higher salt content (6% and 8% NaCl, **Supplementary Figure 2**). This indicated that crab isolate had moderately halophilic bacterium as a previous study defined that moderately halophilic bacterium grew optimally in media containing 3%–15% (w/v) salt (Ventosa et al., 1998).

**Table 1:** Summary of capability of *Vibrio alginolyticus* SWS growth in different salt conditions.

Salt % in broth	Growth
0	–
1	+
6	+
8	+

#### 3.5.2 Antimicrobial susceptibilities

Antimicrobial susceptibility test was conducted to investigate the antimicrobial-resistant profile of *V. alginolyticus* SWS. The antimicrobial-resistant profile of *V. alginolyticus* SWS is illustrated in **Table 2**. V*. alginolyticus* SWS was resistant to two antibiotics, including colistin and ampicillin among 20 tested antibiotics/antibiotic combinations, while sensitive to the remaining 18 antibiotic agents except 6 antibiotics that do not have official interpretation. These 12 antibiotic agents were highly effective in inhibiting *V. alginolyticus* SWS, which were amoxicillin–clavulanic acid (AUG/AMC), cefepime (FEP), cefotaxime (CTX), cefoxitin (FOX), ceftazidime (CAZ), chloramphenicol (C), ciprofloxacin (CIP), gentamycin (CN), imipenem (IMP), meropenem (MEM), tetracyclin (TE), and trimethoprim/sulfamethoxazole (SXT). However, there is no official reference from the European Committee on Antimicrobial Susceptibility Testing and Clinical and Laboratory Standards Institute for the zone of inhibition of ceftriaxone (CRO), ertapenem (EPT), doripenem (DOR), cefotaxime + clavulanic acid (CTL), ceftazidime + clavulanic acid (CAL), and nalidixic acid (NA) (Clinical and Laboratory Standards Institute, 2016).

**Table 2:** Antimicrobial susceptibility test of *Vibrio alginolyticus* SWS.

Antimicrobial agent (μg/disc)	Zone of inhibition (Diameter, mm)	MIC (µg/ml)	Interpretation
Amoxicillin-clavulanic acid (AUG/AMC, 20 + 10 = 30 µg)	32	N.A	S
Ampicillin (AMP, 10 µg)	10	N.A	R
Cefepime (FEP, 30 µg)	31	0.66	S
Cefotaxime (CTX, 30 µg)	33	0.37	S
Cefotaxime + clavulanic acid (CTL, 30 + 10 = 40 µg)	35	N.A	N.A
Cefoxitin (FOX, 30 µg)	24	3.8	S
Ceftazidime (CAZ, 30 µg)	27	1.6	S
Ceftazidime + clavulanic acid (CAL, 30 + 10 = 40 µg)	30	0.88	N.A
Ceftriaxone (CRO, 30 µg)	36	N.A	N.A
Chloramphenicol (C, 30 µg)	36	0.95	S
Ciprofloxacin (CIP, 5 µg)	29	< 0.11	S
Colistin (CS) (10 µg)	11	N.A	R
Doripenem (DOR) (10 µg)	36	N.A	N.A
Ertapenem (EPT) (10 µg)	37	N.A	N.A
Gentamycin (CN,10 µg)	32	< 1.3	S
Imipenem (IMP) (10 µg)	36	<0.56	S
Meropenem (MEM) (10 µg)	37	<0.3	S
Nalidixic acid (NA) (30 µg)	18	2.6	N.A
Tetracyclin (TE) (30 µg)	28	0.42	S
Trimethoprim/sulfamethoxazole (SXT) (25 µg)	33	< 0.21	S

'S' means sensitive to antibiotic; 'R' means resistant; 'MIC' means minimum inhibitory concentration. ‘N.A’ means no official zone of inhibition references from EUCAST and CLSI or MIC is not available.

#### 3.5.3 Metabolic/biochemical characterization

Metabolic/biochemical characterization of *V. alginolyticus* SWS was evaluated by Vitek 2 GN cards. The summary of metabolic/biochemical characteristics are shown in **Table 3**. V*. alginolyticus* SWS was identified as *V. alginolyticus* and had positive results on 11 biochemical tests, including ala-phe-pro-arylamidase (APPA), L-pyrrolydonyl-arylamidase (PyrA), beta-n-acetyl-glucosaminidase (BNAG), phosphatase (PHOS), glycine arylamidase (GlyA), coumarate (CMT), Glu-Gly-Arg-arylamidase (GGAA), D-glucose (dGLU), fermentation/glucose (OFF), L-proline arylamidase (ProA), and tyrosine arylamidase (TyrA).

**Table 3:** Summary of metabolic and physiological characterization of isolated *Vibrio alginolyticus* SWS reported in this study and other *V. alginolyticus* strains in other studies.

Biochemical Test (mnemonic) (mg/well)	Strain name			
	*V. alginolyticus SWS* (With 0.45% Saline)	Vibrio seto (With 0.85% Saline)	*V. alginolyticus* ATCC 17749 (Ben Abdallah et al., 2023)	*V. alginolyticus* MK170250 (Emam et al., 2019)
Ala-phe-pro-arylamidase (APPA) (0.0384)	+	+	N.A	N.A
Adonitol (ADO) (0.1875)	–	–	N.A	N.A
L-pyrrolydonyl-arylamidase (PyrA) (0.018)	+	+	N.A	N.A
L-arabitol (IARL) (0.3)	–	–	N.A	N.A
D-cellobiose (dCEL) (0.3)	–	–	N.A	N.A
Beta-galactosidase (BGAL) (0.036)	–	–	–	N.A
H_2_S production (H_2_S) (0.0024)	–	–	N.A	N.A
beta-n-acetyl- glucosaminidase (BNAG) (0.0408)	+	+	–	N.A
Glutamyl arylamidase pNA (AGLTp) (0.0324)	–	–	N.A	N.A
D-glucose (dGLU) (0.3)	+	+	+	+
Gamma-glutamyl-transferase (GGT) (0.0228)	–	–	N.A	N.A
Fermentation/glucose (OFF) (0.45)	+	+	N.A	N.A
Beta-glucosidase (BGLU) (0.036)	–	–	–	N.A
D-maltose (dMAL) (0.3)	–	–	N.A	N.A
D-mannitol (dMAN) (0.1875)	–	–	N.A	+
D-mannose (dMNE) (0.3)	–	–	N.A	N.A
Beta-xylosidase (BXYL) (0.0324)	–	–	N.A	N.A
Beta-alanine arylamidase pNA (BAlap) (0.0174)	–	–	N.A	N.A
L-proline arylamidase (ProA) (0.0234)	+	+	N.A	N.A
Lipase (LIP) (0.0192)	–	–	+	N.A
Palatinose (PLE) (0.3)	–	–	N.A	N.A
Tyrosine arylamidase (TyrA) (0.0276)	+	+	N.A	N.A
Urease (URE) (0.15)	–	–	N.A	–
D-sorbitol (dSOR) (0.1875)	–	–	N.A	+
Saccharose/sucrose (SAC) (0.3)	–	+	N.A	+
D-tagatose (dTAG) (0.3)	–	–	N.A	N.A
D-trehalose (dTRE) (0.3)	–	+	N.A	N.A
Citrate (CIT) (0.054)	–	–	–	+
Malonate (MNT) (0.15)	–	–	N.A	N.A
5-Keto-d-gluconate (5KG) (0.3)	–	–	N.A	N.A
L-lactate alkalinization (ILATk) (0.15)	–	+	N.A	N.A
Alpha-glucosidase (AGLU) (0.036)	–	–	–	N.A
Succinate alkalinization (SUCT) (0.15)	–	+	N.A	N.A
Beta-N-acetyl- galactosaminidase (NAGA) (0.0306)	–	–	N.A	N.A
Alpha-galactosidase (AGAL) (0.036)	–	–	–	N.A
Phosphatase (PHOS) (0.0504)	+	+	N.A	N.A
Glycine arylamidase (GlyA) (0.012)	+	–	N.A	N.A
Ornithine decarboxylase (ODC) (0.3)	–	–	N.A	+
Lysine decarboxylase (LDC) (0.15)	–	–	N.A	+
L-histidine assimilation (IHISa) (0.087)	–	–	N.A	N.A
Coumarate (CMT) (0.126)	+	+	N.A	N.A
Beta-glucuronidase (BGUR) (0.0378)	–	–	–	N.A
O/129 resistance (O129R) (0.0105)	–	–	N.A	N.A
Glu-Gly-Arg-arylamidase (GGAA) (0.0576)	+	+	N.A	N.A
L-malate assimilation (IMLTa) (0.042)	–	–	N.A	N.A
Ellman (ELLM) (0.03)	–	+	N.A	N.A
L-lactate assimilation (ILATa) (0.186)	–	–	N.A	N.A

'+' means positive result; '-' negative result; ‘N.A’ means this study did not include these parameter(s).

#### 3.5.4 Optimal temperature and pH

**Figure 7:**
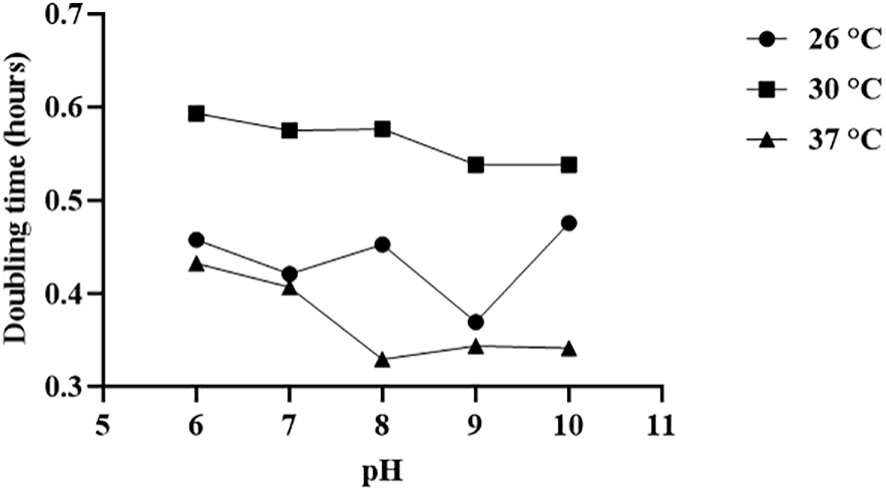
Optimal temperature and pH for the growth of *Vibrio alginolyticus* SWS. The optimal temperature for the growth of *Vibrio alginolyticus SWS* is 37°C, while the optimal pH for the growth of *V. alginolyticus* SWS depended on the temperature. Generally, the isolate grew better at the pH range of 8–10.

The growth kinetics of *V. alginolyticus* SWS at different pH and temperature, in terms of doubling time, was determined (**Figure 7**). The optimal temperature for the growth of this strain is 37°C with the lowest doubling times at pH 6–10, while the doubling times at 26°C were slightly longer than that, followed by those at 30°C. On the other hand, the isolate failed to grow at pH 4 at all tested temperatures, and the growth at pH 5 was insufficient to achieve the stationary phase within 14 h, with the OD600 values below 1.0 (data not shown). From pH 6–10, the optimal pH for the growth of the isolate depended on the temperature. Generally, the isolate grew better at a pH range of 8–10. At 37°C, the optimal pH range was 8–10 since the doubling times at these pH values were similar and generally lower than those at pH 6–7. When the temperature decreased to 30°C, the doubling times at pH 9–10 were lower than those at pH 6–8. Yet, the doubling times at 26°C fluctuated as the pH increased. While the optimal pH is 9, the doubling time increased significantly when the pH increased to 10.

## 4 Discussion

The demand for mud crab is an increasing trend due to its high nutritional value and palatability. However, increasing the supply of mud crab in aquaculture are mainly constrained by cannibalism and disease outbreak (Liew et al., 2023). Unlike cannibalism that can be easily managed via improving knowledge and experience of the farmer, outbreak of diseases remains the biggest challenge in mud crab aquaculture. This is because the diseases can result in high mortality with no effective treatment, and some diseases have causative pathogens that may be unknown (Coates and Rowley, 2022). In addition, the use of antibiotics is not recommended in aquaculture because of the concern of developing antibiotic resistance bacteria eventually affecting humans (Hoseinifar et al., 2024). Hence, the investigation of the disease outbreaks in mud crab aquaculture to formulate effective preventative control measures for preventing the recurrence of disease outbreaks is important to maintain the sustainability of mud crab aquaculture. In this study, *V. alginolyticus* SWS was isolated from local crab aquaculture responsible for causing high morality, which showed high pathogenicity in experimental infection of *S. serrata*.

There is only a single report showing that *V. alginolyticus* could cause shell disease in mud crabs, but experimental infection was not used to validate it, and the mortality rate and pathogenicity as well as characteristics were not investigated in detail (Gunasekaran et al., 2019). Based on the current understanding of mud crab diseases, they are mainly caused by pathogens, including mud crab reovirus, mud crab dicistrovirus, mud crab tombus-like virus (MCTV), *V. parahaemolyticus*, and *Aquimarina hainanensis* (Coates and Rowley, 2022). Furthermore, there has been an increase in outbreaks of disease causing high mortality in aquatic animals, for instance, fish, oysters, and shrimp associated with *V. alginolyticus* (George et al., 2005; Cao et al., 2018; Yang et al., 2021), with unknown mortality in crabs. Therefore, *V. alginolyticus* could be an emerging pathogen in mud crab aquaculture. Our result suggested that the disease outbreak accounted for 70% of mortality resulting from *V. alginolyticus* infection, which leads to excessive economic loss to the aquaculture.

Experimental infection has been commonly employed to validate whether the isolated causative agents in diseases meet the criteria of Koch’s postulates and to investigate its pathogenicity (Xie et al., 2021). Therefore, this study also performed experimental infection to determine whether *V. alginolyticus* SWS was a novel causative agent in the recent local massive death of *S. serrata* aquaculture. *V. alginolyticus* SWS was only reisolated from experimentally infected *S. serrata* (**Figures 1C-F**), and the same clinical symptoms were observed in infected mud crabs from farm and experimental infected *S. serrata* implying that *V. alginolyticus* SWS was the causative agent. Results from experimental infection showed that *V. alginolyticus* SWS could result in high mortality (75%) after 4 days of infection. Previously, *V. alginolytigus* was found to cause shell disease in *S. serrata* with obvious shell lesions or induce whitish muscle syndrome (Gunasekaran et al., 2019; Hou et al., 2023). However, *V. alginolyticus* SWS did not induce these symptoms; instead, it caused inactivity, low appetite, and decolorization of the hepatopancreas, gills, and opaque muscle. Furthermore, in the present study, 2.5 × 10^5^ CFU/ml of *V. alginolyticus* SWS induced 75% mortality after 4 days of infection in *S. serrata*, while other studies required much higher CFU of *V. alginolytigus* to induce a similar mortality rate in oysters and shrimps, at 5 × 10^7^ and 4 × 10^8^ CFU/ml, respectively (Chun-Hung et al., 2004; Yang et al., 2021). This indicated that *S. serrata* were more vulnerable than oysters and shrimps to *V. alginolytigus* probably because it affected different parts of mud crabs. The occurrence of *V. alginolytigus* SWS outbreak in mud crabs might increase.

Histopathology was employed to understand the negative impact of *V. alginolyticus* SWS on mud crabs. The significant histological differences in the hepatopancreas, gills, and muscles between healthy and infected mud crabs provided clues about how the infection of this new isolate of *Vibrio* caused the death of mud crabs. Some typical histological features indicated inflammation in the tissues observed, such as eosinophilia and infiltration of hemocytes. Targeting pathogens, hemocyte plays a crucial role in the innate immunity of crustaceans (Zhang et al., 2019). As infiltration of hemocytes was only seen in the hepatopancreas and gills of the infected crabs, but not in the muscles, it was possible that the infection primarily affects the former two organs, while the muscle was subjected to the secondary impact of infection. Besides, various cellular features suggested that the tissues also experienced necrosis of different extents, including nuclear rounding, pyknosis, and loss of histological architecture. Heavy necrosis in the three types of tissues suggested that the infection could cause systemic damage to the organs of the crabs. The histological observation of the hepatopancreas and muscles resonates with previous studies (Xie et al., 2021; John et al., 2024). For example, in the infection of *S. paramamosain* by *Photobacterium* sp., similar pathologies were observed in the hepatopancreas with tissue necrosis, widened lumen, and detachment of epithelial cells from basement membrane (Xie et al., 2021), while the muscles fibers were disorganized, and similar muscular atrophy was observed (Xie et al., 2021). When *Photobacterium* species infects shrimps, similar hepatopancreatic damage was seen with widened lumen and damaged epithelial cells (Liu et al., 2016). Besides, in the co-infection of *S. serrata* by mud crab reovirus and *Staphylococcus saprophyticus*, hemocyte infiltration and degradation of interstitial tissues were also seen. The lumen of the hepatopancreatic tubules reduced in size, which was also observed in the less infected crabs (John et al., 2024). The extent of damage in the present study was larger compared to these previous studies suggesting a high virulence of this strain that might be due to its virulence factor-related genes.

Molecular identification of bacteria is commonly performed by 16S rRNA gene sequencing; however, 16S sequencing is unable to identify the species level between *Vibrio* species due to a high similarity in the 16S rRNA region of their genomes (Mougin et al., 2020). A recent study misidentified species and strains in *Vibrio* species because of using 16S rRNA sequencing only or multilocus sequence analysis (MLSA) of four genes. The same study suggested at least performing MLSA of eight genes or even more advanced techniques to achieve reliable identification up to the strain level (Jiang et al., 2021). In this study, we first tentatively identified the *V. alginolyticus* SWS isolated from natural infected crab by MALDI-TOF MS as *V. alginolytigus*, then WGS was performed and submitted to the PATRIC server for comprehensive genome analysis to confirm the species. The genome similarity of *V. alginolyticus* SWS was also calculated by ANI using JSpeciesWS.

Regarding the metabolic/biochemical characteristics, our study aligned with the findings of a previous study of *V. alginolyticus ATCC 17749* showing that *V. alginolyticus* SWS could not grow in salt-free environment (Farid and Larsen, 1981). Here, in our study, we found that some of the metabolic/biochemical characteristics of *V. alginolyticus* SWS were different from those of *V. alginolyticus* ATCC 17749 that has been reported to be positive to lipase, negative to beta-n-acetyl- glucosaminidase (Ben Abdallah et al., 2023), while *V. alginolyticus* SWS was also found to be different from *V. alginolyticus* MK170250 isolated from stingray (Emam et al., 2019), which had positive result on lysine decarboxylase, ornithine decarboxylase, citrate, mannitol, and D-sorbitol.

Antibiotic resistance in *vibrio* species is a major obstacle in the prevention and management of disease outbreak that can result in huge economic loss. A recent study reported that 43 out of 104 V*. alginolyticus* isolates from diseased aquaculture in China exhibited multiple antimicrobial resistance to at least four kinds of antibiotics, in which all of them are resistant to ampicillin (Yu et al., 2022). Our findings were aligned with the previous study, which also showed that *V. alginolyticus* SWS is resistant to ampicillin. These results could provide fundamentals for designing prevention and management control of the occurrence of *V. alginolyticus* SWS in aquaculture, particularly mud crab. Ideally, alternatives should be considered rather than using antibiotics such as using Chinese herbal medicines that have served as a good replacement for antibiotics to treat bacterial infection in mud crabs (Wu et al., 2021).

## 5 Conclusion

In summary, our study identified that *V. alginolyticus* SWS was responsible for a recent outbreak in *S. serrata* aquaculture with validation on experiential infection. Results from experimental infection and histopathological analysis implied that *V. alginolyticus* SWS was highly pathogenic and lethal to *S. serrata*. Genome features of *V. alginolyticus* SWS and its antimicrobial resistance (AMR) genes and virulence factor-related genes were also studied in our study by comprehensive genome analysis and were predicted in a virulence factor database. Our results also showed that *V. alginolyticus SWS* was clustered to *V. alginolyticus* ATCC 17749, but some of the metabolic/biochemical characteristics were different. Antimicrobial susceptibility tests in this study indicated that *V. alginolyticus* SWS was resistant to ampicillin. The discovery of the emerging pathogen *V. alginolyticus* SWS in *S. serrata* in this study highlights the importance of identifying emerging pathogens and formulating feasible management to reduce economic loss in mud crab agriculture.

## Data availability statement

The datasets presented in this study can be found in online repositories. The names of the repository/repositories and accession number(s) can be found in the article/**Supplementary Material**.

## Ethics statement

The use of invertebrates in research or teaching activities does not require ethical approval from ethics committee. The studies were conducted in accordance with the local legislation and institutional requirements.

## Author contributions

CK: Data curation, Formal analysis, Investigation, Methodology, Writing – original draft. RCWY: Data curation, Formal analysis, Investigation, Methodology, Writing – original draft. PTH: Data curation, Methodology, Writing – review & editing. KC: Data curation, Methodology, Writing – review & editing. IN: Data curation, Formal analysis, Methodology, Writing – review & editing. JF: Data curation, Formal analysis, Methodology, Writing – review & editing. IW: Data curation, Formal analysis, Methodology, Writing – review & editing. MCYY: Data curation, Formal analysis, Methodology, Writing – review & editing. WML: Writing – review & editing. HKK: Methodology, Resources, Supervision, Validation, Writing – review & editing. GKHS: Methodology, Project administration, Resources, Supervision, Writing – review & editing. FWNC: Conceptualization, Methodology, Project administration, Resources, Supervision, Writing – review & editing. SWS: Conceptualization, Funding acquisition, Project administration, Resources, Supervision, Writing – review & editing.

## Supplementary material

Supplementary Figure 1 Results of the comprehensive genome analysis of *Vibrio alginolyticus* SWS **(A)** Circular graphical assembly of the genome of *Vibrio alginolyticus* SWS, from outer to inner rings, the contigs, CDS on the forward strand, CDS on the reverse strand, RNA genes, CDS with homology to known antimicrobial resistance genes, CDS with homology to known virulence factors, GC content and GC skew; The colours of the CDS on the forward and reverse strand indicate the subsystem (refer to B) that these genes belong to. **(B)** Subsystem analysis of the genome of *Vibrio alginolyticus* SWS.

Supplementary Figure 2 Salt-tolerant of *Vibrio alginolyticus* SWS in different percentage of salt in nutrient broth. *V. alginolyticus* SWS could not grow in NB with no salt, in contrast, *V. alginolyticus* SWS grew in NB ranging from 1% to 8% NaCl.
