# Supplementary figures and images for: Characteristics and pathogenicity of Vibrio alginolyticus SWS causing high mortality in mud crab (Scylla serrata) aquaculture in Hong Kong
Source: Front Cell Infect Microbiol. 2024 Jul 23;14:1425104. doi: 10.3389/fcimb.2024.1425104 (PMC11300173; doi:10.3389/fcimb.2024.1425104)

## Slide 1
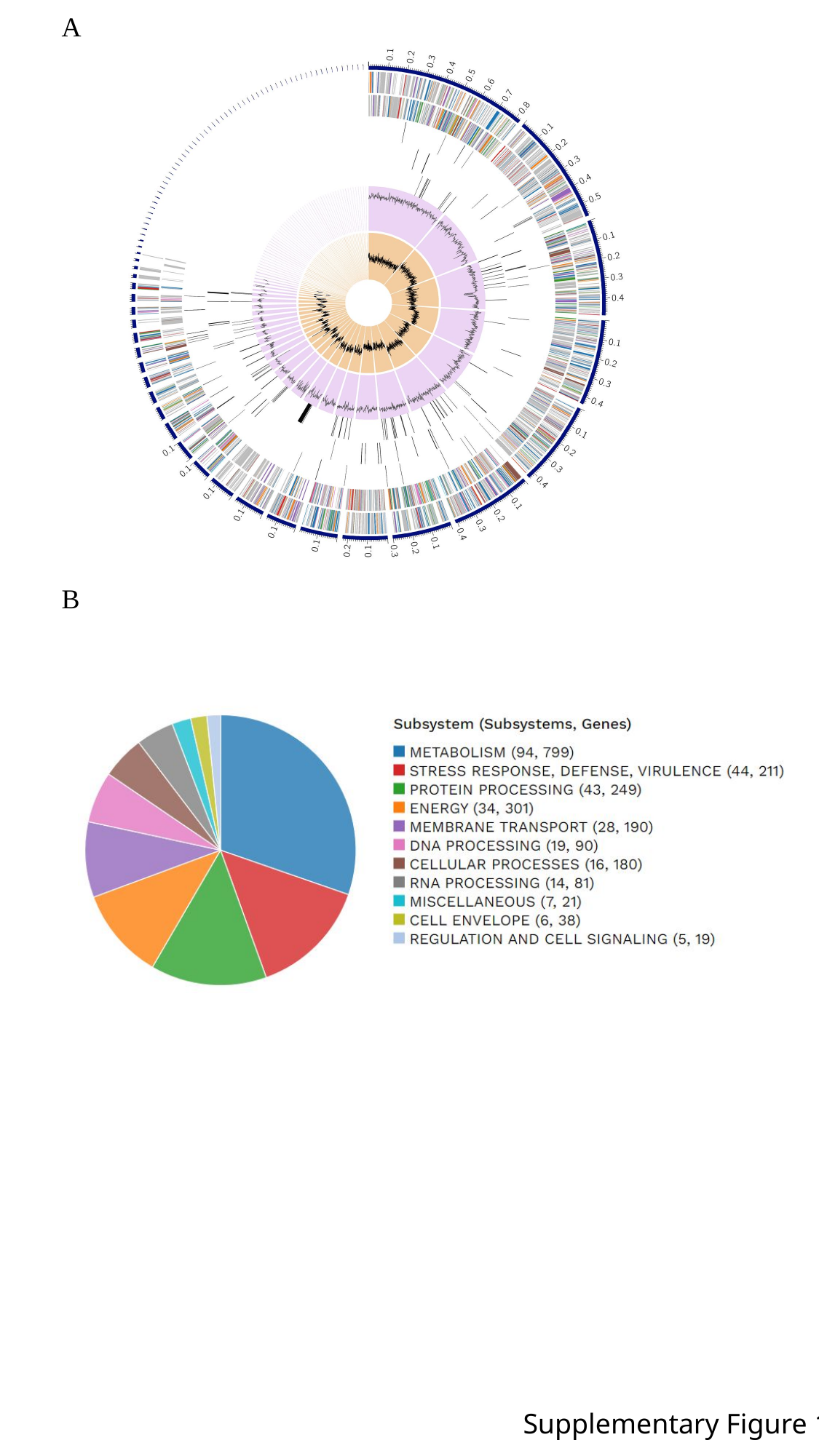

A
B
Supplementary Figure 1

## Slide 2
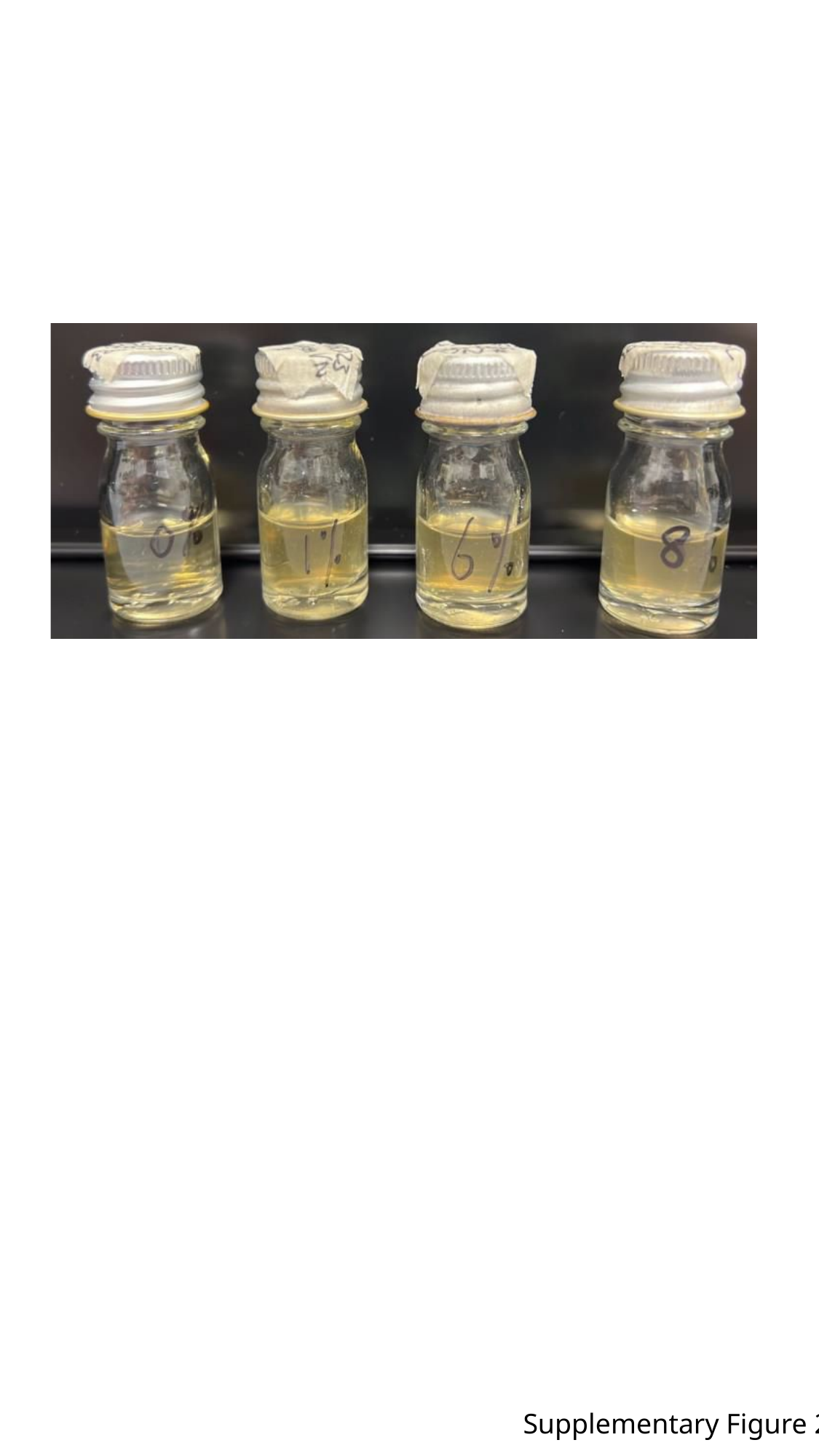

#
Supplementary Figure 2

Supplement: Supplementary Figure 1 — Results of the comprehensive genome analysis of Vibrio alginolyticus SWS (A) Circular graphical assembly of the genome of Vibrio alginolyticus SWS, from outer to inner rings, the contigs, CDS on the forward strand, CDS on the reverse strand, RNA genes, CDS with homology to known antimicrobial resistance genes, CDS with homology to known virulence factors, GC content and GC skew; The colours of the CDS on the forward and reverse strand indicate the subsystem (refer to B) that these genes belong to. (B) Subsystem analysis of the genome of Vibrio alginolyticus SWS. [file Presentation_1.pptx]
