# Supplementary material for: Characteristics and pathogenicity of Vibrio alginolyticus SWS causing high mortality in mud crab (Scylla serrata) aquaculture in Hong Kong
Source: Front Cell Infect Microbiol. 2024 Jul 23;14:1425104. doi: 10.3389/fcimb.2024.1425104 (PMC11300173; doi:10.3389/fcimb.2024.1425104)
Supplement: Supplementary file 2 [file Table_1.docx]

| Supplementary table 1. Assembled and annotated genome features of *Vibrio alginolyticus* SWS | |
| --- | --- |
| Feature | Value |
| Number of contigs | 87 |
| Genome Size (bp) | 5,215,511 |
| GC content | 44.61% |
| Contig N50 | 433,558 bp |
| Contig L50 | 5 |
| Number of Chromosomes | 0 |
| Number of protein coding sequences (CDS) | 4,876 |
| Number of transfer RNA (tRNA) genes | 78 |
| Number of ribosomal RNA (rRNA) genes | 3 |
| Number of partial CDS genes | 0 |
| Number of miscellaneous RNA genes | 0 |
| Number of repeat regions | 0 |

| Supplementary table 2. Protein features of *Vibrio alginolyticus* SWS | |
| --- | --- |
| **Feature** | **Number** |
| Hypothetical proteins | 1128 |
| Proteins with functional assignments | 3748 |
| Proteins with EC number assignments | 1087 |
| Proteins with GO assignments | 910 |
| Proteins with Pathway assignments | 802 |
| Proteins with PATRIC genus-specific family (PLfam) assignments | 4636 |
| Proteins with PATRIC cross-genus family (PGfam) assignments | 4694 |

| Supplementary table 3. Specialty antimicrobial resistance (AMR) genes present in *Vibrio alginolyticus* SWS | |
| --- | --- |
| **AMR** **mechanism** | **Genes** |
| Antibiotic activation enzyme | *KatG* |
| Antibiotic inactivation enzyme | *CARB* family |
| Antibiotic target in susceptible species | *Alr, Ddl, dxr, EF-G, EF-Tu, folA, Dfr, folP, gyrA, gyrB, Iso-tRNA, kasA, MurA, rho, rpoB, rpoC, S10p, S12p* |
| Antibiotic target protection protein | *QnrB* family |
| Antibiotic target replacement protein | *fabV* |
| Efflux pump conferring antibiotic resistance | *MacA, MacB, MdtL, Tet(35), TolC/OpmH* |
| Gene conferring resistance via absence | *gidB* |
| Protein altering cell wall charge conferring antibiotic resistance | *GdpD, PgsA* |
| Regulator modulating expression of antibiotic resistance genes | *H-NS, OxyR* |

| Supplementary table 4. Virulence Factors related genes in *Vibrio alginolyticus* SWS | | | |
| --- | --- | --- | --- |
| **VF class** | **Virulence factors** | **Related genes** | ***V. alginolyticus* SWS** |
| Adherence | Accessory colonization factor | *acfA* | - |
|  |  | *acfB* | - |
|  |  | *acfC* | - |
|  |  | *acfD* | - |
|  | Mannose-sensitive hemagglutinin (MSHA type IV pilus) | *mshA* | + |
|  |  | *mshB* | - |
|  |  | *mshC* | + |
|  |  | *mshD* | + |
|  |  | *mshE* | + |
|  |  | *mshF* | + |
|  |  | *mshG* | + |
|  |  | *mshH* | + |
|  |  | *mshI* | + |
|  |  | *mshJ* | + |
|  |  | *mshK* | + |
|  |  | *mshL* | + |
|  |  | *mshM* | + |
|  |  | *mshN* | + |
|  | Toxin-coregulated pilus (type IVB pilus) | *tcpA* | - |
|  |  | *tcpB* | - |
|  |  | *tcpC* | - |
|  |  | *tcpD* | - |
|  |  | *tcpE* | - |
|  |  | *tcpF* | - |
|  |  | *tcpH* | - |
|  |  | *tcpI* | - |
|  |  | *tcpJ* | - |
|  |  | *tcpN/toxT* | - |
|  |  | *tcpP* | - |
|  |  | *tcpQ* | - |
|  |  | *tcpR* | - |
|  |  | *tcpS* | - |
|  |  | *tcpT* | - |
|  | Type IV pilus | *pilA* | + |
|  |  | *pilB* | + |
|  |  | *pilC* | + |
|  |  | *pilD* | + |
| Antiphagocytosis | Capsular polysaccharide | *cpsA* | + |
|  |  | *cpsB* | + |
|  |  | *cpsC* | + |
|  |  | *cpsD* | + |
|  |  | *cpsE* | + |
|  |  | *cpsF* | + |
|  |  | *cpsG* | + |
|  |  | *cpsH* | + |
|  |  | *cpsI* | + |
|  |  | *cpsJ* | + |
|  |  | *hp1* | - |
|  |  | *rmlA* | - |
|  |  | *rmlB* | - |
|  |  | *rmlC* | - |
|  |  | *rmlD* | - |
|  |  | *wbfB* | - |
|  |  | *wbfC* | - |
|  |  | *wbfT* | - |
|  |  | *wbfU* | - |
|  |  | *wbfV/wcvB* | + |
|  |  | *wbfY* | - |
|  |  | *wbjD/wecB* | - |
|  |  | *wbuB* | - |
|  |  | *wcaJ* | - |
|  |  | *wecA* | + |
|  |  | *wecC* | - |
|  |  | *wza* | + |
|  |  | *wzb* | + |
|  |  | *wzc* | + |
|  | Capsule(Klebsiella) | *uge* | + |
| Chemotaxis and motility | Flagella | *cheA* | + |
|  |  | *cheB* | + |
|  |  | *cheR* | + |
|  |  | *cheV* | + |
|  |  | *cheW* | + |
|  |  | *cheY* | + |
|  |  | *cheZ* | + |
|  |  | *filM* | + |
|  |  | *flaA* | + |
|  |  | *flaB* | + |
|  |  | *flaC* | - |
|  |  | *flaD* | - |
|  |  | *flaE* | + |
|  |  | *flaG* | + |
|  |  | *flaI* | + |
|  |  | *flgA* | + |
|  |  | *flgB* | + |
|  |  | *flgC* | + |
|  |  | *flgD* | + |
|  |  | *flgE* | + |
|  |  | *flgF* | + |
|  |  | *flgG* | + |
|  |  | *flgH* | + |
|  |  | *flgI* | + |
|  |  | *flgJ* | + |
|  |  | *flgK* | + |
|  |  | *flgL* | + |
|  |  | *flgM* | + |
|  |  | *flgN* | + |
|  |  | *flhA* | + |
|  |  | *flhB* | + |
|  |  | *flhF* | + |
|  |  | *flhG* | + |
|  |  | *fliA* | + |
|  |  | *fliD* | + |
|  |  | *fliE* | + |
|  |  | *fliF* | + |
|  |  | *fliG* | + |
|  |  | *fliH* | + |
|  |  | *fliI* | + |
|  |  | *fliJ* | + |
|  |  | *fliK* | + |
|  |  | *fliL* | + |
|  |  | *fliN* | + |
|  |  | *fliO* | + |
|  |  | *fliP* | + |
|  |  | *fliQ* | + |
|  |  | *fliR* | + |
|  |  | *fliS* | + |
|  |  | *flrA* | + |
|  |  | *flrB* | + |
|  |  | *flrC* | + |
|  |  | *motA* | + |
|  |  | *motB* | + |
|  |  | *motX* | + |
|  |  | *motY* | + |
| Enzyme | Metalloproteinase | *hap/vvp* | - |
|  | Neuraminidase | *nanH* | - |
| Iron uptake | Enterobactin receptors | *irgA* | + |
|  |  | *vctA* | + |
|  | Heme receptors | *hasR* | - |
|  |  | *hutA* | + |
|  |  | *hutR* | + |
|  | Periplasmic binding protein-dependent ABC transport systems | *vctC* | + |
|  |  | *vctD* | + |
|  |  | *vctG* | + |
|  |  | *vctP* | + |
|  |  | *viuC* | - |
|  |  | *viuD* | - |
|  |  | *viuG* | - |
|  |  | *viuP* | - |
|  | Vibriobactin | *vibA* | - |
|  |  | *vibB* | - |
|  |  | *vibC* | - |
|  |  | *vibD* | - |
|  |  | *vibE* | - |
|  |  | *vibF* | - |
|  |  | *vibH* | - |
|  |  | *viuA* | - |
|  |  | *viuB* | - |
| Quorum sensing | Autoinducer-2 | *luxS* | + |
|  | Cholerae autoinducer-1 | *cqsA* | + |
| Secretion system | EPS type II secretion system | *epsC* | + |
|  |  | *epsE* | + |
|  |  | *epsF* | + |
|  |  | *epsG* | + |
|  |  | *epsH* | + |
|  |  | *epsI* | + |
|  |  | *epsJ* | + |
|  |  | *epsK* | + |
|  |  | *epsL* | + |
|  |  | *epsM* | + |
|  |  | *epsN* | + |
|  |  | *gspD* | + |
|  | T3SS1 secreted effectors | *vopQ* | + |
|  |  | *vopR* | + |
|  |  | *vopS* | + |
|  | T3SS1 | *sycN* | + |
|  |  | *tyeA* | + |
|  |  | *vcrD* | + |
|  |  | *vcrG* | + |
|  |  | *vcrH* | + |
|  |  | *vcrR* | + |
|  |  | *vcrV* | + |
|  |  | *virF* | + |
|  |  | *virG* | + |
|  |  | *vopB* | + |
|  |  | *vopD* | + |
|  |  | *vopN* | + |
|  |  | *vscA* | + |
|  |  | *vscB* | + |
|  |  | *vscC* | + |
|  |  | *vscD* | + |
|  |  | *vscF* | + |
|  |  | *vscG* | + |
|  |  | *vscH* | + |
|  |  | *vscI* | + |
|  |  | *vscJ* | + |
|  |  | *vscK* | + |
|  |  | *vscL* | + |
|  |  | *vscN* | + |
|  |  | *vscO* | + |
|  |  | *vscP* | - |
|  |  | *vscQ* | + |
|  |  | *vscR* | + |
|  |  | *vscS* | + |
|  |  | *vscT* | + |
|  |  | *vscU* | + |
|  |  | *vscX* | + |
|  |  | *vscY* | + |
|  |  | *vxsC* | + |
|  | T3SS2 secreted effectors | *vopA* | - |
|  |  | *vopC* | - |
|  |  | *vopL* | - |
|  |  | *vopT* | - |
|  | T3SS2 | *vcrD2* | - |
|  |  | *vscC2* | - |
|  |  | *vscN2* | - |
|  | VAS effector proteins | *hcp-1* | - |
|  |  | *hcp-2* | - |
|  |  | *vgrG-1* | - |
|  |  | *vgrG-2* | - |
|  |  | *vgrG-3* | - |
|  | VAS type VI secretion system | *vasA* | - |
|  |  | *vasB* | - |
|  |  | *vasC* | - |
|  |  | *vasD* | - |
|  |  | *vasE* | - |
|  |  | *vasF* | - |
|  |  | *vasG* | - |
|  |  | *vasH* | - |
|  |  | *vasI* | - |
|  |  | *vasJ* | - |
|  |  | *vasK* | - |
|  | T4SS effectors(Coxiella) |  | + |
| Toxin | Accessory cholera enterotoxin | *ace* | - |
|  | Cholera toxin | *ctxA* | - |
|  |  | *ctxB* | - |
|  | Hemolysin/cytolysin | *vvhA* | - |
|  | RTX toxin | *rtxA* | - |
|  |  | *rtxB* | - |
|  |  | *rtxC* | - |
|  |  | *rtxD* | - |
|  | Thermolabile hemolysin | *tlh* | + |
|  | Thermostable direct hemolysin | *tdh* | - |
|  | *V.cholerae* cytolysin | *hlyA* | - |
|  | Zonula occludens toxin | *zot* | - |
|  | Phytotoxin coronatine (*Pseudomonas*) |  | + |
|  | Phytotoxin phaseolotoxin (*Pseudomonas*) | *cysC1* | + |
| Biofilm formation | AdeFGH efflux pump/transport autoinducer (*Acinetobacter*) | *adeG* | + |

+ and – indicates presence or absence of the VF gene respectively.

|  | Supplementary table 5. Genome similarity of *Vibrio alginolyticus* SWS | | | | | | | | | | | | | | | |
| --- | --- | --- | --- | --- | --- | --- | --- | --- | --- | --- | --- | --- | --- | --- | --- | --- |
|  | ANIb (ANIm) | | | | | | | | | | | | | | | |
| Genome | 1 | 2 | 3 | 4 | 5 | 6 | 7 | 8 | 9 | 10 | 11 | 12 | 13 | 14 | 15 | 16 |
| 1. *V. alginolyticus* SWS | * | 98.67  (98.84) | 98.67  (98.84) | 98.41  (98.64) | 98.37 (98.56) | 98.27  (98.54) | 98.41  (98.58) | 98.39  (98.61) | 98.42  (98.60) | 98.42  (98.60) | 98.42  (98.59) | 98.38  (98.58) | 98.41 (98.63) | 98.28 (98.51) | 98.42 (98.60) | 98.40 (98.58) |
| 2. *V. alginolyticus* BSW15 | 98.72 (98.84) | * | 99.98 (99.99) | 98.38 (98.63) | 98.43 (98.59) | 98.41 (98.61) | 98.46 (98.59) | 98.37 (98.57) | 98.44 (98.57) | 98.44 (98.58) | 98.47 (98.59) | 98.38 (98.59) | 98.41 (98.61) | 98.26 (98.51) | 98.48 (98.61) | 98.46 (98.59) |
| 3. *V. alginolyticus* BSW8 | 98.68  (98.84) | 99.98 (99.99) | * | 98.39 (98.63) | 98.40 (98.58) | 98.36 (98.61) | 98.42 (98.60) | 98.39 (98.56) | 98.40 (98.57) | 98.40 (98.58) | 98.44 (98.59) | 98.36 (98.59) | 98.38 (98.61) | 98.23 (98.52) | 98.45 (98.61) | 98.43 (98.59) |
| 4.*V. alginolyticus* V1 | 98.41 (98.64) | 98.43 (98.64) | 98.43 (98.63) | * | 98.36 (98.60) | 98.30 (98.57) | 98.38 (98.59) | 98.45 (98.60) | 98.36 (98.59) | 98.36 (98.60) | 98.39 (98.59) | 98.32 (98.60) | 98.34 (98.61) | 98.24 (98.51) | 98.38 (98.60) | 98.38 (98.58) |
| 5. *V. alginolyticus* RM-10-2 | 98.30 (98.56) | 98.36 (98.58) | 98.36 (98.58) | 98.28 (98.60) | * | 98.20 (98.52) | 98.42 (98.62) | 98.33 (98.60) | 98.44 (98.58) | 98.44 (98.59) | 98.43 (98.61) | 98.26 (98.53) | 98.36 (98.57) | 98.21 (98.49) | 98.42 (98.64) | 98.42 (98.61) |
| 6. *V. alginolyticus* RM-12-1 | 98.18 (98.54) | 98.27 (98.61) | 98.27 (98.61) | 98.17 (98.57) | 98.10 (98.51) | * | 98.16 (98.52) | 98.16 (98.54) | 98.15 (98.52) | 98.14 (98.52) | 98.17 (98.52) | 98.45 (98.70) | 98.10 (98.52) | 98.01 (98.47) | 98.11 (98.54) | 98.14 (98.53) |
| 7. *V. alginolyticus* NCTC12160 | 98.48 (98.59) | 98.50  (98.60) | 98.50 (98.60) | 98.39 (98.60) | 98.52 (98.62) | 98.30 (98.52) | * | 98.40 (98.57) | 98.48 (98.59) | 98.48 (98.60) | 100.00 (99.97) | 98.35 (98.54) | 98.51 (98.60) | 98.32 (98.47) | 99.97 (99.99) | 99.97  (99.94) |
| 8. *V. alginolyticus* UCD-32C | 98.31 (98.61) | 98.30 (98.57) | 98.29 (98.57) | 98.31 (98.60) | 98.32 (98.60) | 98.21 (98.54) | 98.28 (98.57) | * | 98.32 (98.59) | 98.31 (98.60) | 98.29 (98.57) | 98.30 (98.61) | 98.25 (98.59) | 98.14 (98.52) | 98.28 (98.60) | 98.27 (98.57) |
| 9. *V. alginolyticus* UCD-53C | 98.38 (98.60) | 98.36 (98.57) | 98.36 (98.57) | 98.28 (98.59) | 98.45 (98.58) | 98.23 (98.52) | 98.40 (98.59) | 98.34 (98.59) | * | 99.98 (99.98) | 98.41 (98.58) | 98.33 (98.58) | 98.42 (98.66) | 98.17 (98.48) | 98.40 (98.61) | 98.40 (98.59) |
| 10. *V. alginolyticus* UCD-9C | 98.27 (98.61) | 98.27 (98.58) | 98.27 (98.58) | 98.17 (98.60) | 98.33 (98.58) | 98.14 (98.52) | 98.30 (98.60) | 98.25 (98.60) | 99.87 (99.98) | * | 98.31 (98.60) | 98.22 (98.59) | 98.31 (98.66) | 98.07 (98.48) | 98.30 (98.61) | 98.29 (98.60) |
| 11. *V. alginolyticus* FDAARGOS 97 | 98.46 (98.58) | 98.49 (98.59) | 98.49 (98.59) | 98.36 (98.60) | 98.50 (98.61) | 98.27 (98.52) | 99.99 (99.97) | 98.37 (98.57) | 98.46 (98.58) | 98.46 (98.59) | * | 98.33 (98.54) | 98.51 (98.61) | 98.30 (98.47) | 99.95 (99.99) | 99.97 (99.94) |
| 12. *V. alginolyticus* UCD-30C | 98.06 (98.58) | 98.09 (98.60) | 98.11 (98.59) | 98.01 (98.59) | 98.01 (98.53) | 98.29 (98.71) | 98.01 (98.54) | 98.07 (98.61) | 98.07 (98.58) | 98.06 (98.59) | 98.01 (98.54) | * | 98.06 (98.59) | 97.91 (98.50) | 98.00 (98.56) | 97.99 (98.54) |
| 13. *V. alginolyticus* 12G01 | 98.48  (98.62) | 98.49 (98.61) | 98.49 (98.61) | 98.39 (98.61) | 98.49 (98.57) | 98.30 (98.52) | 98.53 (98.61) | 98.34 (98.59) | 98.55 (98.66) | 98.55 (98.66) | 98.53 (98.60) | 98.43 (98.59) | * | 98.34 (98.50) | 98.54 (98.63) | 98.52 (98.60) |
| 14. *V. alginolyticus* 40B | 98.34 (98.51) | 98.29 (98.51) | 98.29 (98.52) | 98.27 (98.51) | 98.30 (98.50) | 98.17 (98.47) | 98.31 (98.47) | 98.26 (98.51) | 98.27 (98.48) | 98.26 (98.48) | 98.32 (98.47) | 98.25 (98.50) | 98.26 (98.50) | * | 98.31 (98.48) | 98.31 (98.47) |
| 15. *V. alginolyticus* NBRC 15630 = ATCC 17749 NBRC 15630 | 98.48 (98.60) | 98.50 (98.61) | 98.50 (98.61) | 98.36 (98.61) | 98.55 (98.64) | 98.29 (98.54) | 99.99 (99.99) | 98.40 (98.60) | 98.49 (98.60) | 98.49 (98.61) | 99.99 (99.99) | 98.37 (98.56) | 98.49 (98.62) | 98.33 (98.48) | * | 99.99 (99.98) |
| 16. *V. alginolyticus* NBRC 15630 = ATCC 17749 | 98.45 (98.58) | 98.48 (98.59) | 98.49 (98.59) | 98.37 (98.59) | 98.50 (98.61) | 98.30 (98.53) | 99.97 (99.94) | 98.38 (98.57) | 98.47 (98.58) | 98.47 (98.59) | 99.98 (99.94) | 98.34 (98.54) | 98.48 (98.60) | 98.30 (98.47) | 99.96 (99.98) | * |

* indicates ANI for Same *vibrio alginolyticus* strains, value represent ANI calculated by BLAST+ (ANIb), while (value) calculated by MUMmer (ANIm)
